# The absence of Pitx3 results in postnatal loss of dopamine neurons and is associated with an increase in the pro-apoptotic Bcl2 factor *Noxa* and cleaved caspase 3

**DOI:** 10.1038/s41419-025-07552-w

**Published:** 2025-04-01

**Authors:** Willemieke M. Kouwenhoven, Edward J. Robinson, Daniek Hamberg, Lars von Oerthel, Marten P. Smidt, Lars P. van der Heide

**Affiliations:** Swammerdam Institute for Life Sciences, Molecular Neuroscience Lab, University of Amsterdam, Amsterdam, The Netherlands

**Keywords:** Differentiation, Neuronal development

## Abstract

Mesodiencephalic dopamine neurons (mdDA) of the substantia nigra pars compacta (SNc) and ventral tegmental area (VTA) play critical roles in regulating movement and motivation. Pitx3 is an essential transcription factor required for proper embryonic development and terminal differentiation of mdDA neurons. Although Pitx3 is expressed in every mdDA neuron, its ablation results only in the absence of the SNc, not the VTA. The developmental stage at which the loss of SNc first becomes apparent, as well as the underlying mechanism, remains elusive. Here, we demonstrate, using a Pitx3 knockout GFP knock-in mouse model, that this loss does not occur during embryogenesis but rather postnatally. Quantification of GFP expression revealed a significant reduction in the total number of dopamine neurons at postnatal day 3, but not at embryonic day 14.5, 15.5, and 18.5. Mechanistically this reduction is accompanied by an increase in the number of cleaved caspase 3-positive GFP neurons, suggesting apoptosis. In addition, RT-PCR performed on isolated GFP neurons, one day before the loss of dopamine neurons revealed a notable elevation in the expression of the pro-apoptotic BH3-only factor *Noxa*. Overexpression of Noxa in dopaminergic MN9D cells dose-dependently increases the level of cleaved caspase 3 and the number of propidium iodide-positive cells, indicating that Noxa expression is sufficient to induce cell death in dopamine cells. Additionally, Noxa expression in MN9D cells, combined with a Bax-inhibiting peptide, reduces the number of cleaved caspase 3-positive and propidium iodide-positive cells, further supporting apoptosis as the mechanistic form of cell death. Overall, our study provides insights into the cell death machinery implicated in the loss of dopamine neurons, which may hold relevance for diseases affected by the loss of dopamine neurons such as Parkinson’s disease, where this is a hallmark feature.

## Introduction

Mesodiencephalic dopaminergic neurons (mdDA) form the *substantia nigra* (SNc) and ventral tegmental area (VTA) and are important enforcers of movement and motivation. Embryonic mdDA precursors go through several phases of maturation before they can be considered terminally differentiated. First, the activation of a myriad of transcription factors (e.g., Nurr1, Pitx3, Otx2, Lmx1a/b, En1/2) is required to induce a proper molecular profile that results in the expression of tyrosine hydroxylase (Th), the rate-limiting enzyme in the production of dopamine [1, 2]. At the same time, mdDA neurons travel downwards from their location of birth (the caudal floorplate and floorplate/basalplate boundary), along radial glial cells to the ventral midbrain, after which they migrate laterally along tangential fibers to their destined niche in the SNc or VTA [3–6]. Second, it has become increasingly clear that a population of mdDA neurons is eliminated during development. Programmed cell death in the developing mouse brain visualized by double-labeling of Th and cleaved caspase 3 (CC3), revealed that the wave of apoptosis commences at E14.5, peaks around P0, and then disappears at P14 [7]. A similar study in rats reported two waves of programmed cell death; the first peaking around birth (E20-E22), and the latter around P14 [8]. That second wave of cell death overlaps with the peak of synapse formation within the striatum in rats (between P13-P17) [9]. Ultimately, differentiation, migration, programmed cell death, and synapse elimination coordinate the development of functional dopaminergic neurons. This is supported by the finding that misplaced neurons co-localized with CC3, suggesting they undergo apoptosis [10]. Evidently, research in the naturally occurring processes controlling maturation, survival, and elimination of mdDA neurons is very relevant to the understanding of the etiology of Parkinson’s disease (PD), in which the mdDA neurons of the SNc are lost [11].

A mutant model that has been widely used in mdDA research is the aphakia model. In this model, the homeodomain transcription factor Pitx3, which is present in every mdDA neuron [12], is ablated (either by a double genomic deletion [13, 14] or replaced by GFP [15]), and as a consequence, mdDA neurons of the SNc are lost in the adult brain. Analysis of these genetic models shows that already at E12.5 the expression of Th was reduced [14]. However, as of yet, it is unclear what mechanism is responsible for the selective absence of SNc neurons, and similarly, no consensus exists on the time of death of these neurons, varying between E14.5 and P0 [15, 16].

In this study, we temporally investigated the development of mdDA neurons in control Pitx3GFP/+ and Pitx3GFP/GFP animals to determine exactly at which timepoint the SNc is lost. Utilizing the GFP expression of dopaminergic neurons we determined the number and the location of mdDA neurons along the medial-lateral axis at several developmental stages. Here, we reveal that mdDA neurons in Pitx3-ablated animals are not lost during embryogenic development but postnatally at P3. Prior to this neuronal loss, a subset of GFP-positive mdDA neurons are misplaced evidenced by the presence of a broader area of mdDA neurons in the medial midbrain at E18.5. The loss of neurons at P3 is accompanied by an increase in GFP/CC3 double-positive cells in Pitx3-ablated animals compared to Pitx3GFP/+ heterozygous animals. The mechanism behind the increase in cleaved caspase 3 and the loss of neurons is suggested to be due to the increase in the pro-apoptotic factor Noxa as it was increased quite substantially in the Pitx3 knockout mice. In addition to this, Noxa expression proved sufficient to induce apoptotic cell death in MN9D cells. Overall our study suggests that the loss of Pitx3 results in Noxa-dependent apoptosis resulting in the absence of the SNc in the adult. These mechanistic insights may prove relevant for disorders affected by dopamine neuron loss such as Parkinson’s disease.

## Materials & methods

### Animals

Embryos were isolated at E12.5, E14.5, E15.5, E16.5, and E18.5, considering the morning of detection of the vaginal plug as E0.5. Tissue was isolated at P2 and P3. Pitx3GFP/+ animals were intercrossed with Pitx3GFP/GFP animals in which the *Pitx3* gene is substituted by a GFP allele [15] to breed Pitx3GFP/+ (control) and Pitx3GFP/GFP (KO) littermates. Pitx3GFP/GFP animals were recognized by the shape of the lens, which is malformed in all Pitx3-deficient animals. Samples were not randomized or analyzed blinded. All procedures and experiments were performed according to the guidelines and legislation and with the approval of the Dutch Ethical Committee.

### Fluorescence-activated cell sorting (FACS) and dissection

Midbrains and rostral hindbrains of Pitx3GFP/+ heterozygous mice and Pitx3GFP/GFP homozygous mice were dissected at P2 in L15-5% Fetal Calf Serum (Gibco). Dissociation and sorting of mid/hindbrains were performed as described previously [17]. In short, freshly isolated tissue was dissociated using a Papain dissociation system (Worthington). Cells were sorted on a BD FACS Aria III using previously described settings [17] and collected in Trizol-LS (Invitrogen).

### Real-time quantitative PCR (RT-qPCR)

Relative expression levels of *Noxa* in FACSorted Pitx3GFP/+ and Pitx3GFP/GFP neurons were determined by quantitative real-time qPCR (Lightcycler 480) using the QuantiTect SYBR Green RT PCR Kit (QIAGEN) according to the manufacturer’s instructions. Expression of *Pitx3* was used as genotype control and the expression of *18S* was used for normalization. Primer sequences used were: *Noxa* forward *5*′*-TGCACCGGACATAACTGTGG-3*′; *Noxa* reverse *5*′*-ACTCGTCCTTCAAGTCTGCT-3*′. Amplification products were separated on an agarose gel to confirm product size. Data was analyzed using the ΔΔCt method. Primers were assumed to have comparable efficiency.

### Fluorescent immunohistochemistry

Embryos were fixed in 4% paraformaldehyde in PBS, cryoprotected in 30% sucrose in PBS, and subsequently stored at −80 °C. Sagittal sections (16 μm) were cut on a cryostat, after which they were washed with PBS and blocked in 4% Fetal Calf Serum (FCS) in TBS-T or PBS-T (0.5% Triton). After another wash treatment with PBS, sections were incubated overnight at 4 °C with the primary antibody in TBS-T or PBS-T. Sections were washed three times (PBS) the following morning and incubated for minimally 2 h at room temperature with secondary antibody in PBS, followed by wash treatment with PBS. DAPI staining was performed (1 mg/ml 1:5000) for 5 min, after which sections were washed with PBS. Finally, sections were embedded with Fluorsave. Primary antibodies that were used: chicken anti-GFP (Abcam, 1:1000), rabbit anti-cleaved caspase-3 (Cell Signaling Technologies), 1:200 Secondary antibodies that were used: goat-anti-chicken Alexa 488 (1:1000) and goat-anti-rabbit Alexa 555 (1:000), all Invitrogen.

### Cell culture, transfections & PI staining

The dopaminergic MN9D cell line was a kind gift from Dr. Thomas Perlmann. The MN9D (RRID:CVCL_M067) cell line is not listed as a commonly misidentified cell line by the International Cell Line Authentication Committee. MN9D dopaminergic cells were cultured on poly-D-lysine (Sigma-Aldrich) coated culture dishes in Dulbecco’s Modified Eagle’s Medium (DMEM; Lonza) supplemented with 2 mM L-glutamine (ThermoFisher Scientific), 1× Penicillin/Streptomycin (Pen/Strep; 100 units/ml; ThermoFisher Scientific) and 10% heat-inactivated fetal bovine serum (HiFBS; Biowest). Cells were incubated in a 5% CO2/95% O_2_ atmosphere at 37 °C. A day before the inhibitor and/or stressor experiments, cells were serum-deprived in DMEM containing 2 mM L-glutamine, 1× Pen/Strep, and 0.5% HiFBS to limit growth factor interference. Transfections were performed with lipofectamine 2000 (Invitrogen) according to the manufacturer’s instructions. The Noxa-GFP (Genscript OMu20604C), Noxa-pcDNA (Genscript OMu20604C) and NOXA-GFP plasmid (Genscript OHu27302) plasmids were purchased from Genscript. PI stainings and analyses were performed as described previously [18].

### Western blotting

Western blots were performed as described previously [18] with minor modifications. Primary antibodies used were rabbit anti-GFP (1:5000; Abcam), rabbit anti-mCherry (Biosensis), rabbit anti-cleaved caspase-3 mAb, rabbit anti-Bax, mouse anti-β-actin (1:5000). All antibodies were purchased from Cell Signaling Technologies and used in a 1:1000 dilution unless noted otherwise.

Uncropped westerns are available in the Supplementary File.

### Quantitative analysis

Images were taken with a fluorescent microscope (Leica) using Metamorph software. Using ImageJ software color-combined images were split into single-channel images, and subsequently turned into binary images (using default settings), after which the binary DAPI image was used as an overlay on top of the GFP image (using an image calculator). This creates a binary image of only the cells that were positive for both DAPI and GFP (cellular particles binary image), which can be objectively counted using the “analyze particles” software of ImageJ (Fig. S1). Each embryo was sectioned in 5 adjacent series at 16 μm. All sections were counted. All sections lateral from the retroflexus (RF) were considered “lateral”. All sections medial to, and including the RF were considered “medial”.

### Proximity ligation assay

Cells were fixed in 4% PFA for 10 min, washed with PBS, and permeabilized for 5 min in 0.5% Triton x-100 in PBS. After a quick wash with PBS, the NaveniFlex Cell MR Red kit (Navinci, NC.MR.100 Red) was used according to the manufacturer’s protocol. The primary antibodies mouse anti-Noxa (Santa Cruz SC-56169, 1:200) and rabbit anti-Mcl1 (CST 94296, 1:200) were incubated O/N at 4 °C. Nuclei were stained with DAPI (1:3000) for 5 min, and cells were embedded with Fluorsave.

### Image analysis

The Leica SP8 confocal microscope was used to obtain five z-stacks of images per sample of GFP and PLA signal at 1 µm intervals with a 40× (HC Plan Apo) objective, and representative stacks of images were obtained with a 63× (HC Plan Apo objective) at 0.6 µm intervals.

### Statistical analysis

Experiments were performed at least in triplicate to allow statistical analysis. Values are expressed as means with standard error of the mean. Unpaired, one or two-tailed *t*-tests were used to determine differences between the two conditions. ANOVA was used to determine differences between more than two conditions, followed by either Dunnett’s multiple comparison test to compare various conditions to a control condition or Tukey’s multiple comparison test to compare all conditions. *p* ≤ 0.05 was considered significant and indicated using an asterisk (*p* < 0.05 = *; *p* < 0.01 = **; *p* < 0.001 = ***, *p* < 0.0001= ****).

## Results

### Significant loss of GFP-positive mdDA neurons at P3 in Pitx3-ablated animals

**Fig. 1:**
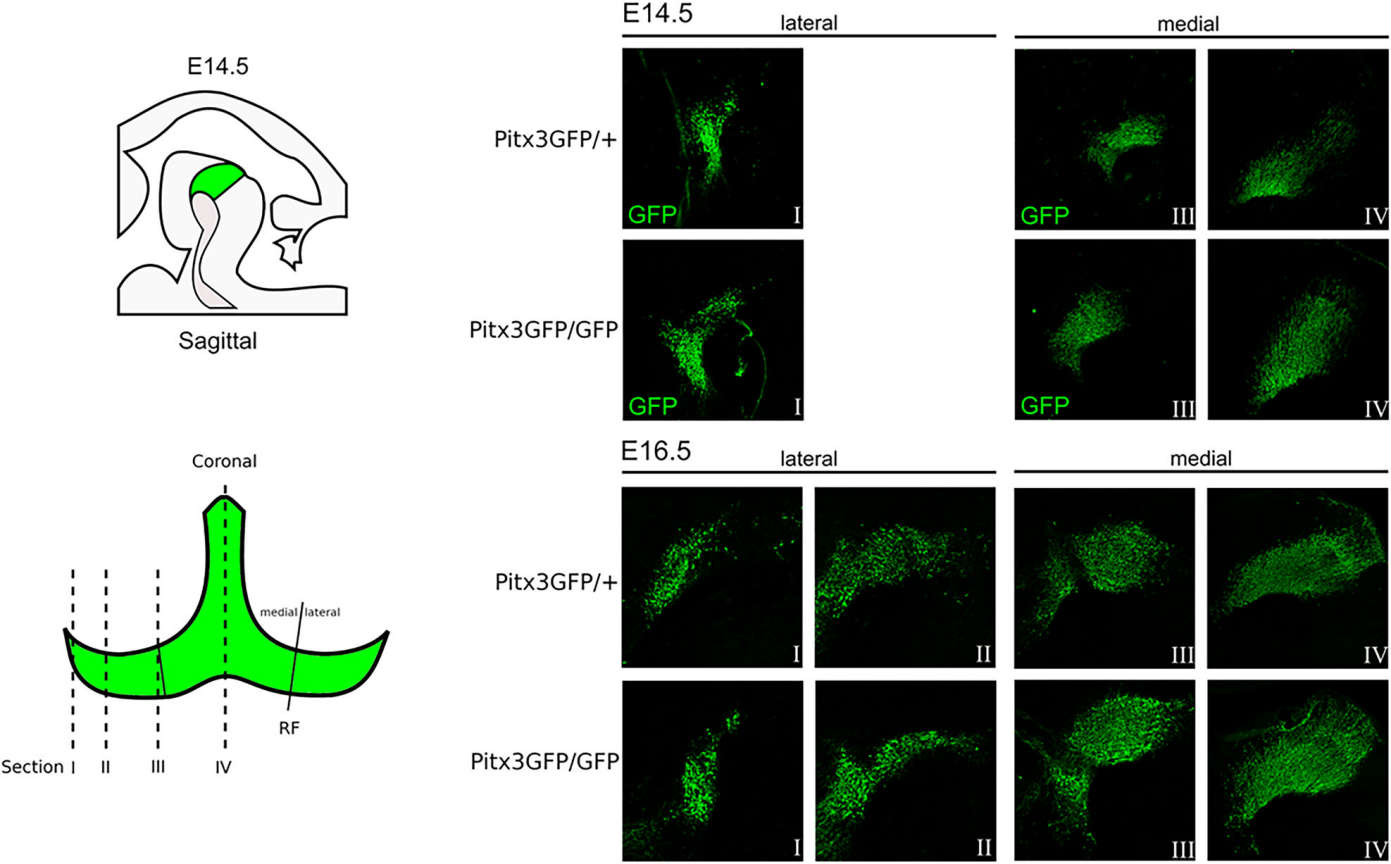
No neuronal loss of mdDA neurons in the absence of Pitx3 at E14,5 and E16,5. (**Left**) Schematic representation of developing mdDA neuronal pool in sagittal and coronal view. In the coronal view, the segregation between lateral (I and II) and medial is indicated (III and IV) (**Right**) GFP immunohistochemistry of lateral sections of Pitx3GFP/+ and Pitx3GFP/GFP animals at E14.5 and E16.5.

**Fig. 2:**
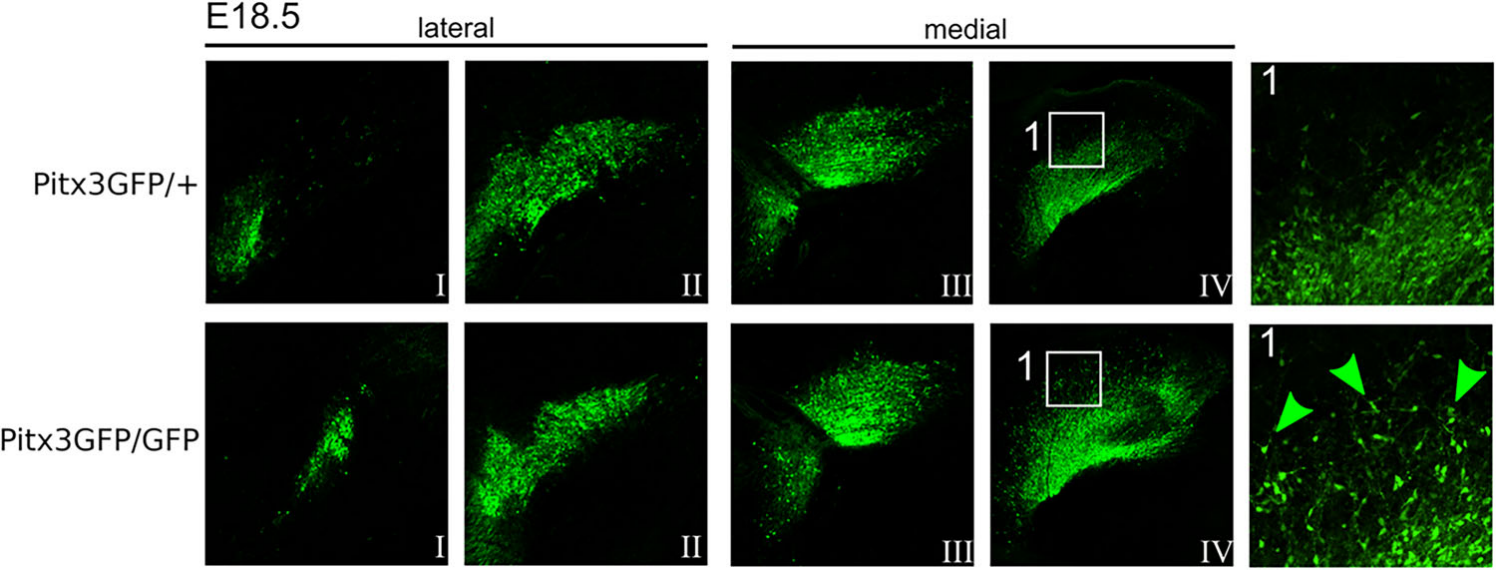
Absence of Pitx3 results in misplaced neurons at E18.5. GFP immunohistochemistry of lateral and medial sections of Pitx3GFP/+ and Pitx3GFP/GFP animals at E18.5. Insets (1) display higher magnification of areas within the white square. Green arrowheads indicate misplaced GFP neurons.

**Fig. 3:**
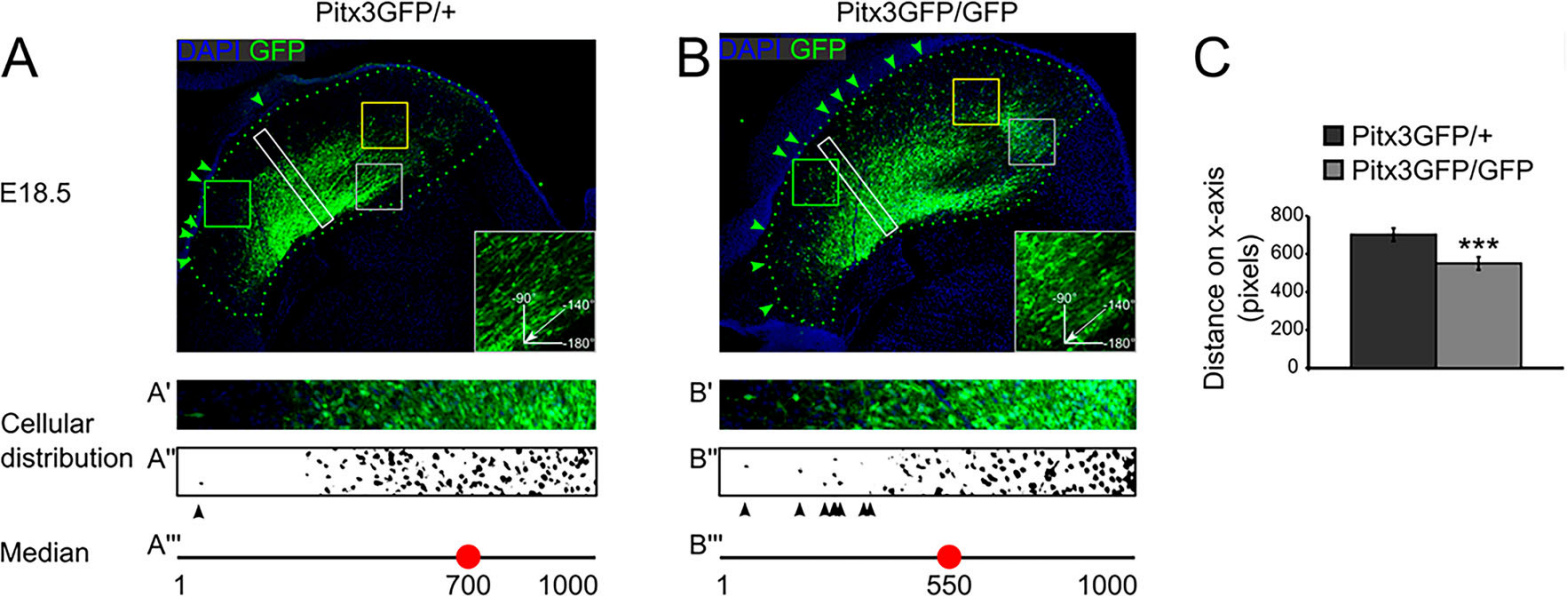
Quantification of misplaced neurons at E18.5. **A** Immunohistochemistry for GFP in the most medial section of the midbrain of Pitx3GFP/+ and Pitx3GFP/GFP animals at E18.5 co-stained for DAPI. The white rectangle of 1000*100 pixels (**A’**) displays the area that was sampled to determine the median location of GFP-positive cells, using the binary image of the overlay of the DAPI and GFP signal (**A”**) which is represented by the red dot (**A’”**). Black arrowheads indicate sporadic GFP-positive neurons close to the ventricular zone in binary image (**A”**). Green dotted line indicates circumference. Green arrowheads indicate sporadic GFP-positive neurons close to the ventricular zone. Inset displays a higher magnification of the gray square and displays the angle of mdDA neurons within this region. **B** The same setup as described in (**A**) for the Pitx3GFP/GFP animal. **C** Quantification of the distribution of GFP-positive neurons as defined in (**A**) and (**B**) in the white rectangle region.

The ablation of the homeodomain transcription factor Pitx3 results in the absence of the SN in the adult mouse. Although a myriad of downstream Pitx3 targets, direct as well as indirect, have been described the mechanistic insight leading to the absence of the SN is lacking. To pinpoint the developmental stage at which the amount of dopamine neurons start diverging between the heterozygous and knockout of Pitx3 we made use of mice that express GFP instead of Pitx3. The Pitx3GFP/GFP were compared to the Pitx3GFP/+ mice as the heterozygote genotype has been shown to develop normally as evidenced by an intact SN [15]. To quantify the amount of dopamine neurons we stained sagittal brain sections at different developmental ages. Subsequently, we stained the sections for GFP in combination with a nuclear DAPI stain. Using an ImageJ semi-automated quantification protocol (see Supplementary Fig. 1), we quantified the amount of GFP expressing cell bodies present. The first developmental stage analyzed was E14.5, a stage which has been described to show loss of well-defined dopamine neuron markers such as Th, Aadc, Drd2, and Vmat2. After the quantification we observed no loss of GFP-positive dopamine neurons in Pitx3GFP/GFP mice as compared to Pitx3GFP/+ mice (Fig. 1). Therefore we subsequently analyzed E16.5 as well as E18.5. Again we observed no significant changes in Pitx3-ablated animals relative to the control (Figs. 1, 2). Having a closer look at the circumvent of the mdDA area in E18.5 Pitx3-ablated animals, it was clear that the GFP-positive mdDA neurons occupy a broader area than in control animals (white box and green arrowheads, Fig. 2). Especially around the medial sections of Pitx3GFP/GFP mice neurons can be observed that reside more distally as compared to the Pitx3GFP/+ mice, suggesting a placement defect (green dotted circumference, Fig. 3A, B). In order to quantify this initial observation we calculated the median of the GFP-positive cell location in the control and Pitx3KO animals (the red dot represents the average median in Fig. 3A”’, B”’). In Pitx3-ablated animals, the median of GFP-positive mdDA neuronal locations was significantly closer to the circumference of the mdDA area (550 pixels ± 34.6 vs 702 pixels ± 34.4, *n* = 3, *p* < 0.01, Fig. 3C). This confirmed our initial observation that in Pitx3-ablated animals GFP-positive mdDA neurons are located in a wider area compared to controls.

**Fig. 4:**
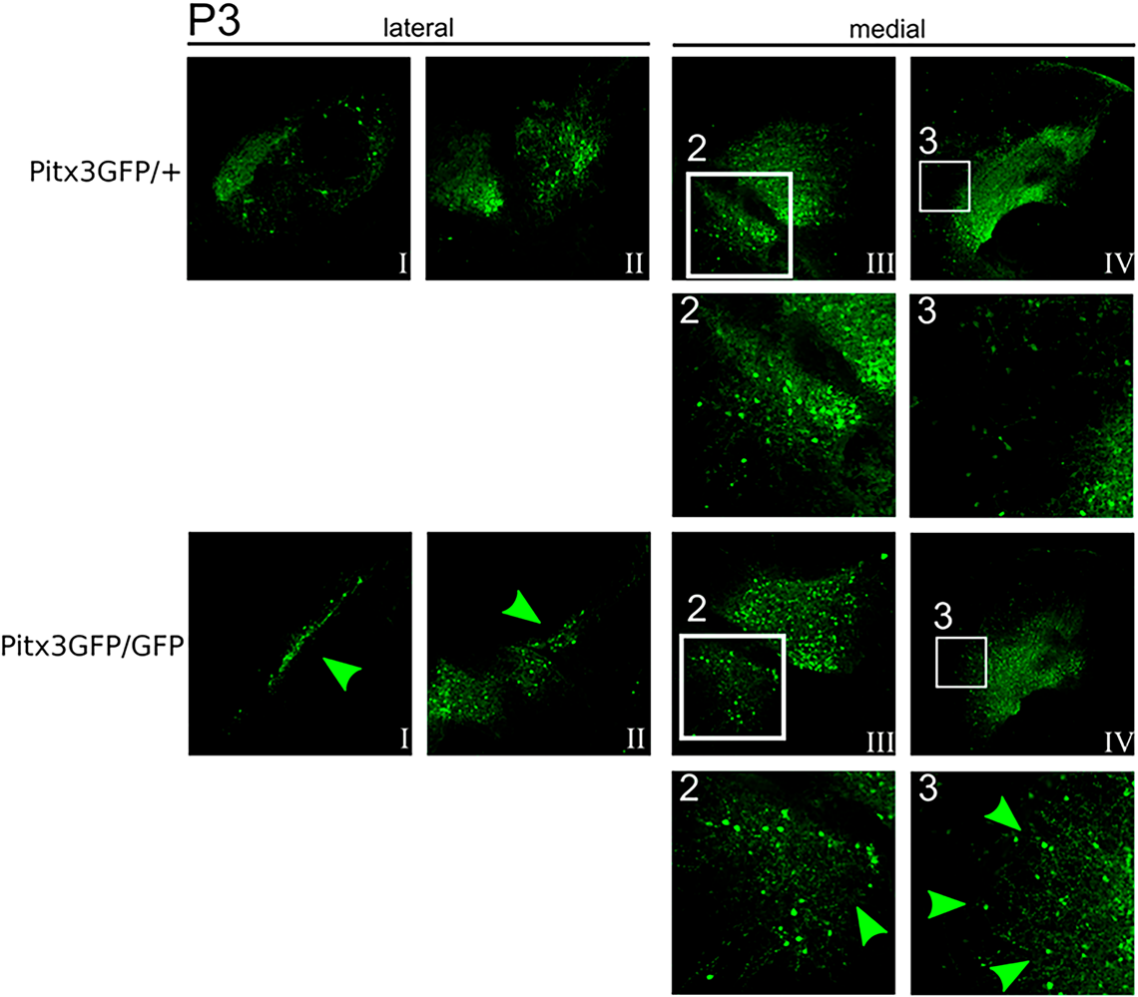
Absence of Pitx3 leads to misplaced neurons and cell loss at P3. GFP immunohistochemistry of lateral and medial sections of Pitx3GFP/+ and Pitx3GFP/GFP animals at Postnatal day 3. Green arrows indicate regions that differ between Pitx3GFP/+ and Pitx3GFP/GFP mice. Insets 2 and 3 display a higher magnification of areas within the white square.

**Fig. 5:**
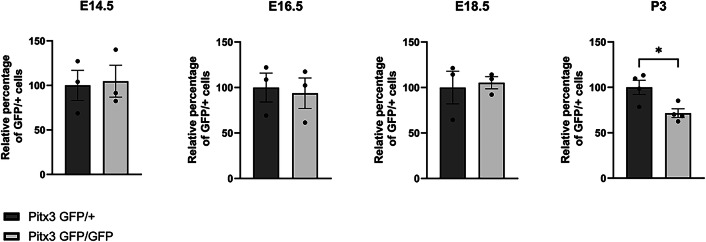
Quantification of GFP-positive cells in sagittal sections in Pitx3GFP/+ and Pitx3GFP/GFP animals from E14.5 to P3. * *n* = 4, *p* < 0.05 one-tailed *t*-test.

Finally, at P3, significantly fewer GFP-positive mdDA neurons were present in the mdDA region compared to the control (100% ± 8.7 vs. 72% ± 4.8, Figs. 4, 5). Analyzing the relative distribution of these GFP-positive neurons on the medial-lateral axis suggested that the loss of GFP-positive mdDA neurons was mainly due to the diminished presence of GFP-positive mdDA neurons in the lateral sections of the midbrain (green arrowheads, Fig. 4).

To assess if the loss of dopamine neurons at P3 was accompanied by the activation of a regulated form of cell death we stained and quantified the amount of neurons positive for cleaved caspase 3 as a marker which may indicate apoptosis.

**Fig. 6:**
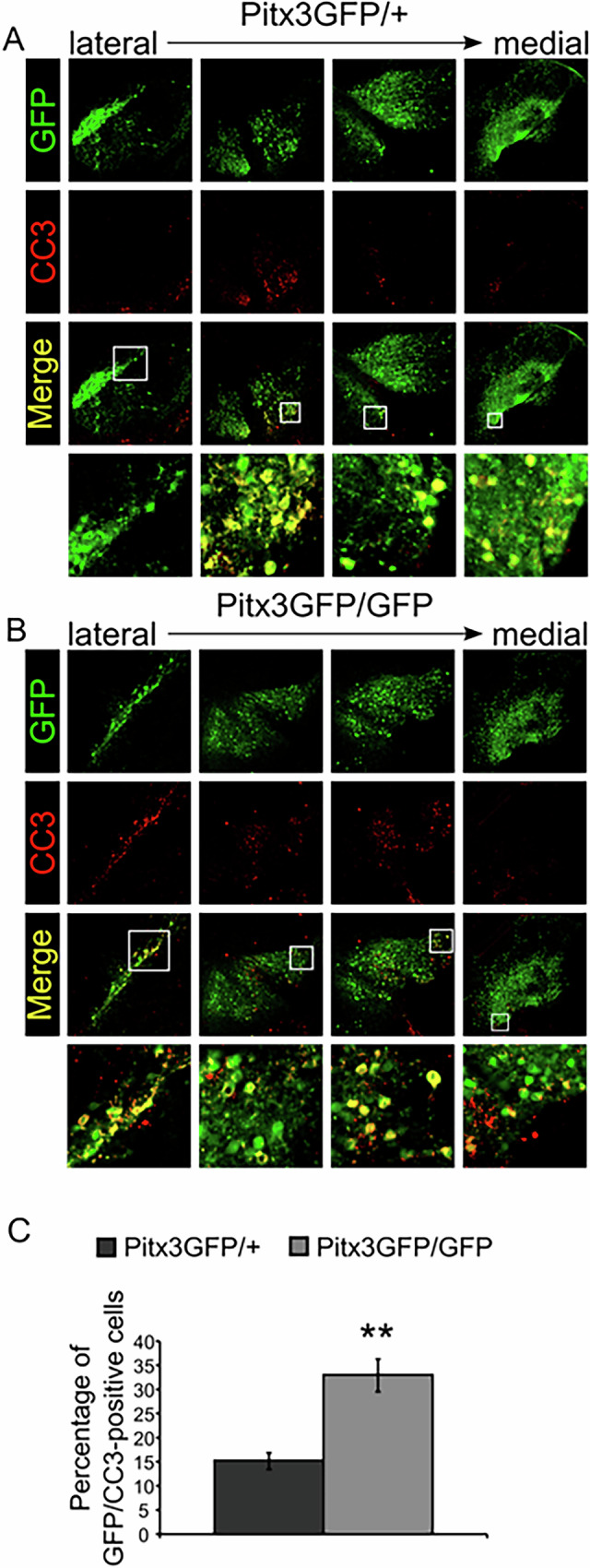
Cleaved caspase 3 is increased in the absence of Pitx3 at P3. **A** Immunohistochemistry for GFP and CC3 in different sections from lateral to medial encompassing the mdDA neuronal pool in Pitx3GFP/+ animals. **B** The same setup as described for **A** in Pitx3GFP/GFP animals (matching sections with **A**). **C** Quantification of GFP/CC3-positive neurons in Pitx3GFP/+ and Pitx3GFP/GFP animals ** *p* < 0.01, *n* = 4.

At P3, 15% ±1.6 of all GFP-positive neurons in control animals are positive for CC3 (Fig. 6A, C) whereas in Pitx3-ablated animals significantly more GFP-positive mdDA neurons are positive for CC3 (33% ± 3.3, Fig. 6B, C). Together, these data demonstrate that at P3, in the absence of Pitx3, already 30% of mdDA neurons are lost (Fig. 5), and of the remaining population another 30% is positive for CC3, suggesting that they undergo programmed cell death, possibly apoptosis.

### *Noxa* mRNA levels are increased in the absence of Pitx3

**Fig. 7:**
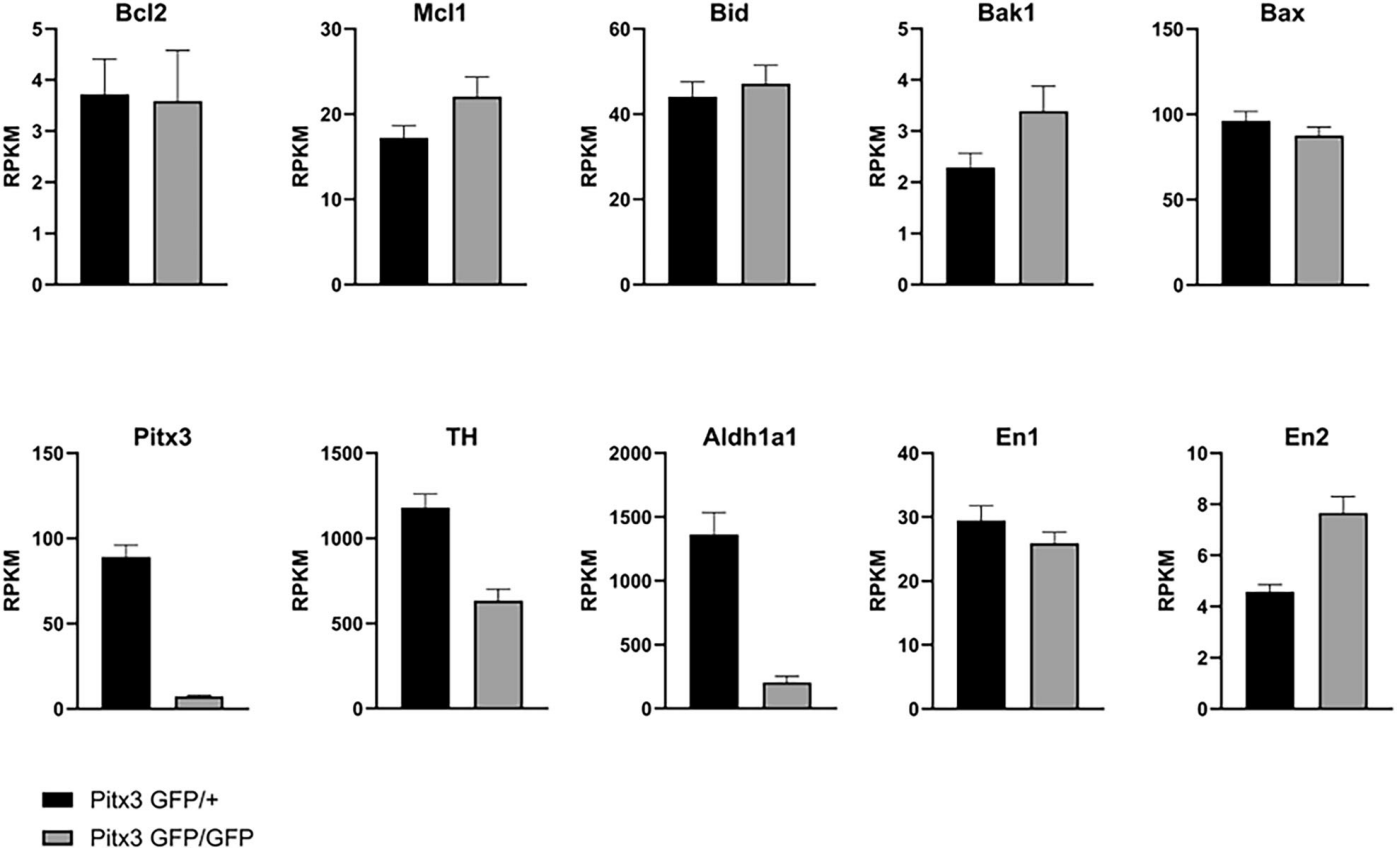
Pitx3 loss leads to changes in the Bcl2 family and Dopaminergic marker transcripts on P1. Reanalysis of scRNA-seq data from Taklova et al. Indicated transcripts are expressed as RPKM. Transcripts with a percentage of zero-reads >90% cells are not shown.

**Fig. 8:**
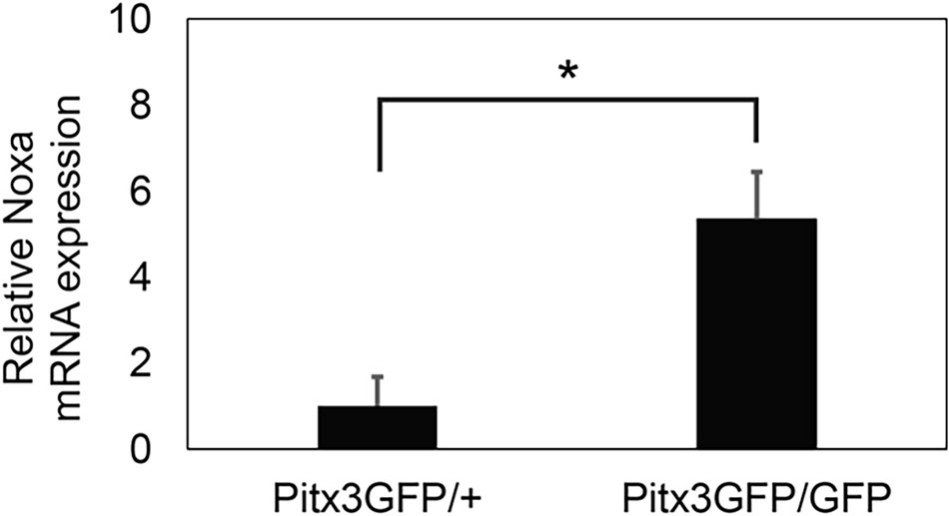
*Noxa* is increased in the absence of Pitx3 at P2. RNA of FACSorted GFP-positive neurons of Pitx3GFP/+ and Pitx3GFP/GFP littermates at P2 was subjected to qPCR with primers directed against Noxa. Relative *Noxa* expression levels are increased in the Pitx3GFP/GFP animals. *p < 0.05, *n* = 10 for Pitx3GFP/+ and *n* = 11 for Pitx3GFP/GFP.

To assess which apoptotic factors are involved in the observed cell loss we reanalyzed data from a study by Tiklova et al., who used the same mouse model to perform single-cell RNA-seq [19]. We chose to examine specific canonical Bcl2 factors as well as described dopamine markers, as positive controls, to be affected by Pitx3. We examined the average expression of all sorted cells combined. Although not all factors could be detected (*Bcl2L10, Bcl2L12, Bcl2L14, Bcl2L15, Bik, Bmf, Pmaip1*), possibly due to the sequence depth, it can clearly be seen that there are subtle changes in Bcl2 factor expression and major changes in dopamine configuration. However, a major Bcl2 factor does not emerge as an obvious candidate (Fig. 7 and Supplementary Fig. 2). Previous work by our lab and others have shown that the anti-apoptotic Bcl2 protein Mcl1 is important for the survival of adult midbrain dopaminergic neurons [18, 20]. In addition, Mcl1 is also of major importance during development, as full knockouts lead to peri-implantation lethality and various conditional knockouts display a high rate of apoptosis [21]. Given the critical role of Mcl1, we hypothesized that upregulation of a BH3-only factor in cells that are not in their appropriate niche possibly inactivates Mcl1 to induce apoptosis. Interestingly, the pro-apoptotic BH3-only protein Noxa has been described as a direct (and indirect) negative regulator of Mcl1 [22–25]. Noxa preferentially binds to Mcl1 over other anti-apoptotic Bcl2 factors such as Bcl2 and Bcl-xL [26]. To that extent, we investigated the expression levels of *Noxa*. GFP-positive neurons from Pitx3GFP/+ heterozygous and Pitx3GFP/GFP homozygous animals were FACSorted at P2 and RNA was isolated from the dopaminergic cells for RT-qPCR experiments with primers directed against *Noxa*. Interestingly, *Noxa* relative expression levels are indeed 4-fold increased in the Pitx3GFP/GFP mouse model compared to Pitx3GFP/+ littermates (100% ± 21 Pitx3GFP/+ vs 430% ± 30 Pitx3GFP/GFP; Fig. 8).

### Noxa overexpression induces apoptosis in dopaminergic MN9D cells

**Fig. 9:**
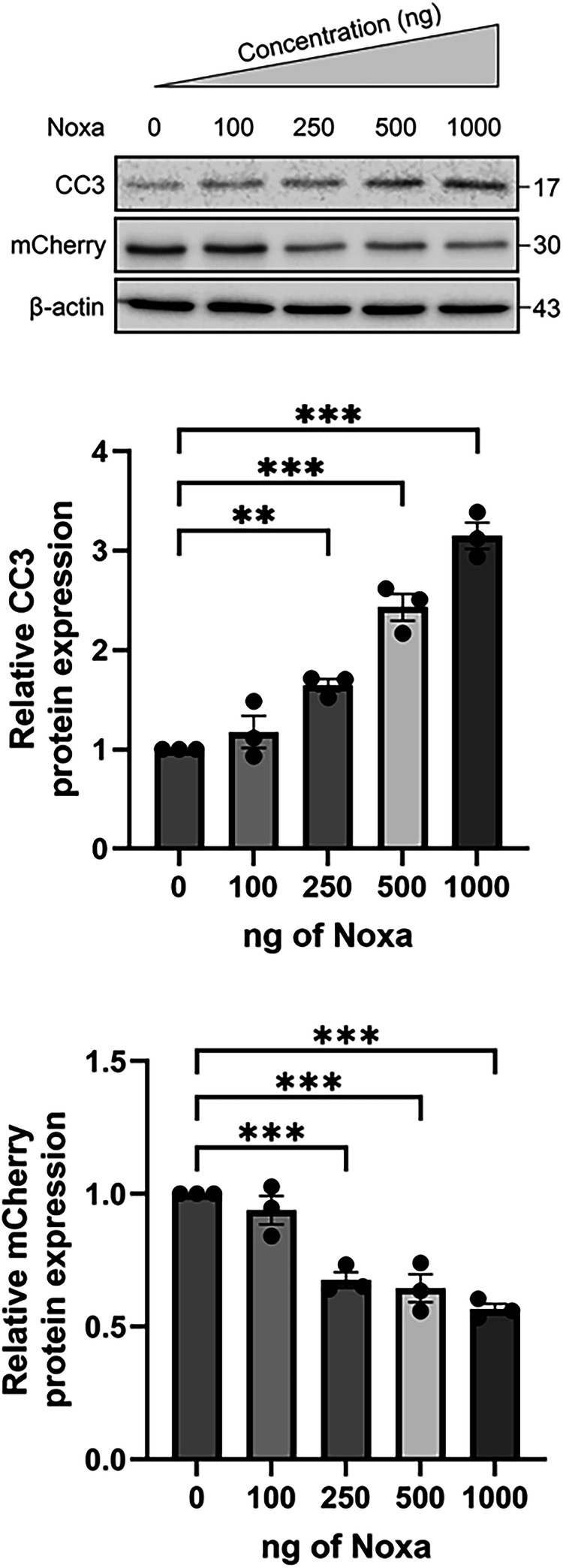
Noxa expression induces cleaved caspase 3 in MN9D cells. Titration of different Noxa concentrations into dopaminergic MN9D cells together with a small amount (100 ng) of EV-mCherry. It is assumed that all mCherry-positive cells also have Noxa transfected. Moreover, MN9D cells do not express Noxa endogenously. Starting from 250 ng Noxa, levels of the apoptosis marker CC3 increase while mCherry levels decrease. This suggests that the introduction of Noxa into MN9D cells induces apoptosis in a dose-dependent manner. **p < 0.01, ***p <  0.001, *n* = 3.

**Fig. 10:**
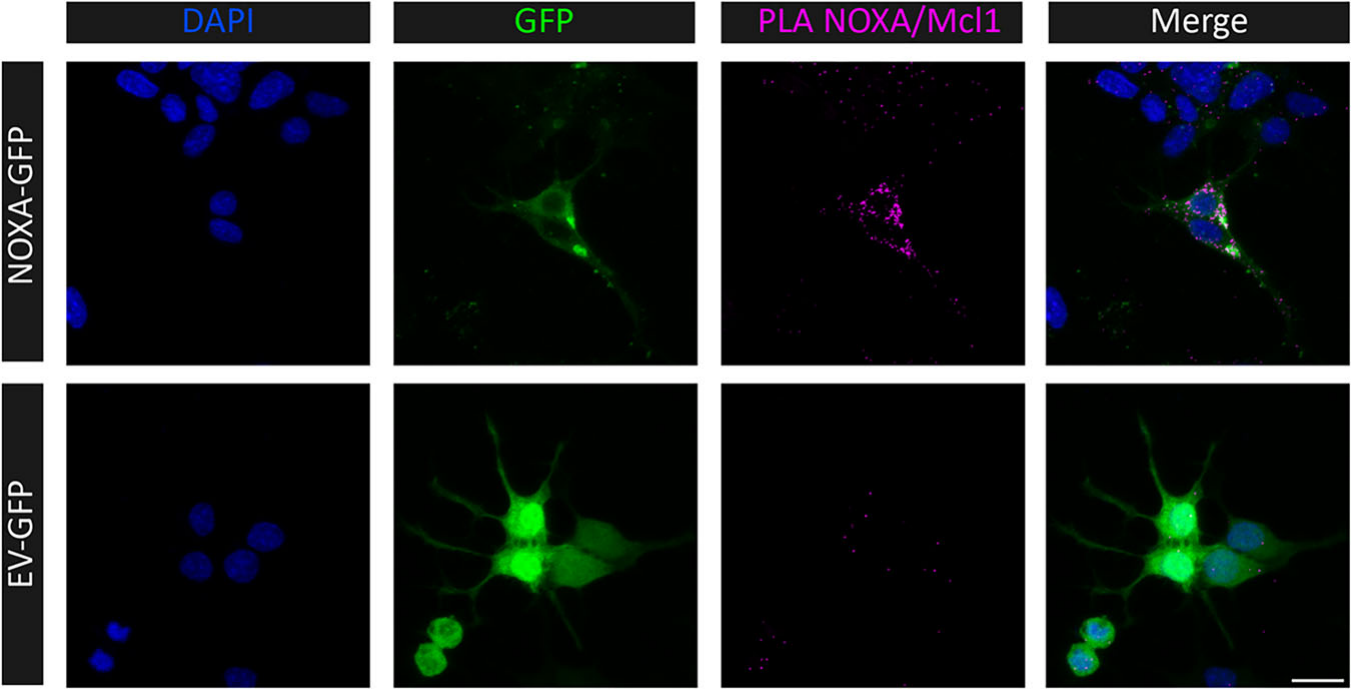
NOXA and Mcl1 interact in MN9D cells. Visualization of NOXA/Mcl1 interactions in MN9D cells overexpressing NOXA-GFP or EV-GFP, where each dot represents a PLA product. MN9D cells lack endogenous expression of Noxa, therefore the EV-GFP condition serves as a negative control. *n* = 5, scalebar 15 µm.

**Fig. 11:**
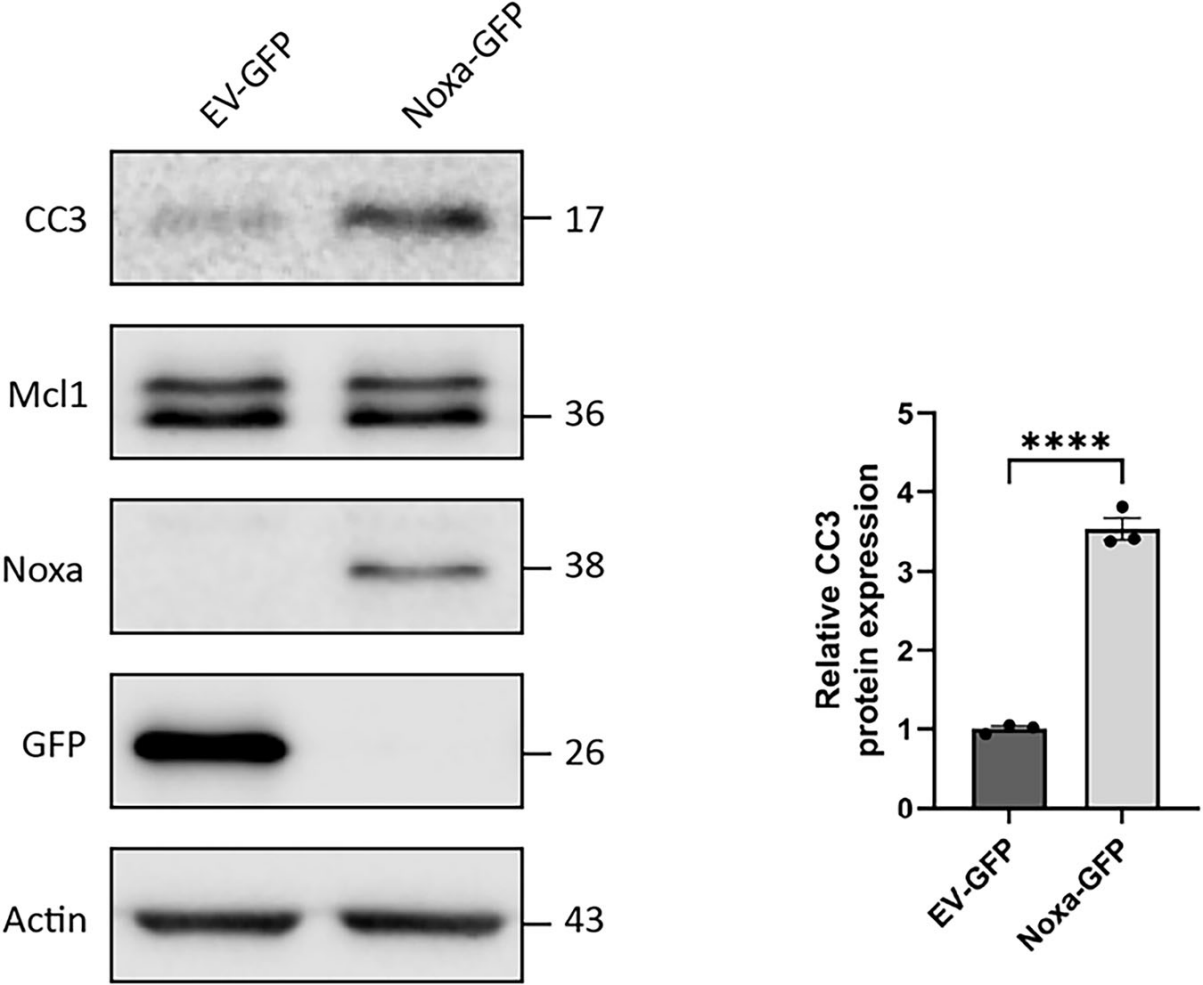
Noxa expression does not alter Mcl1 protein levels. Western blot of MN9D cells overexpressing EV-GFP or Noxa-GFP. Overexpression of Noxa increases CC3 levels while Mcl1 remains unchanged. ****p < 0.0001, *n* = 3.

**Fig. 12:**
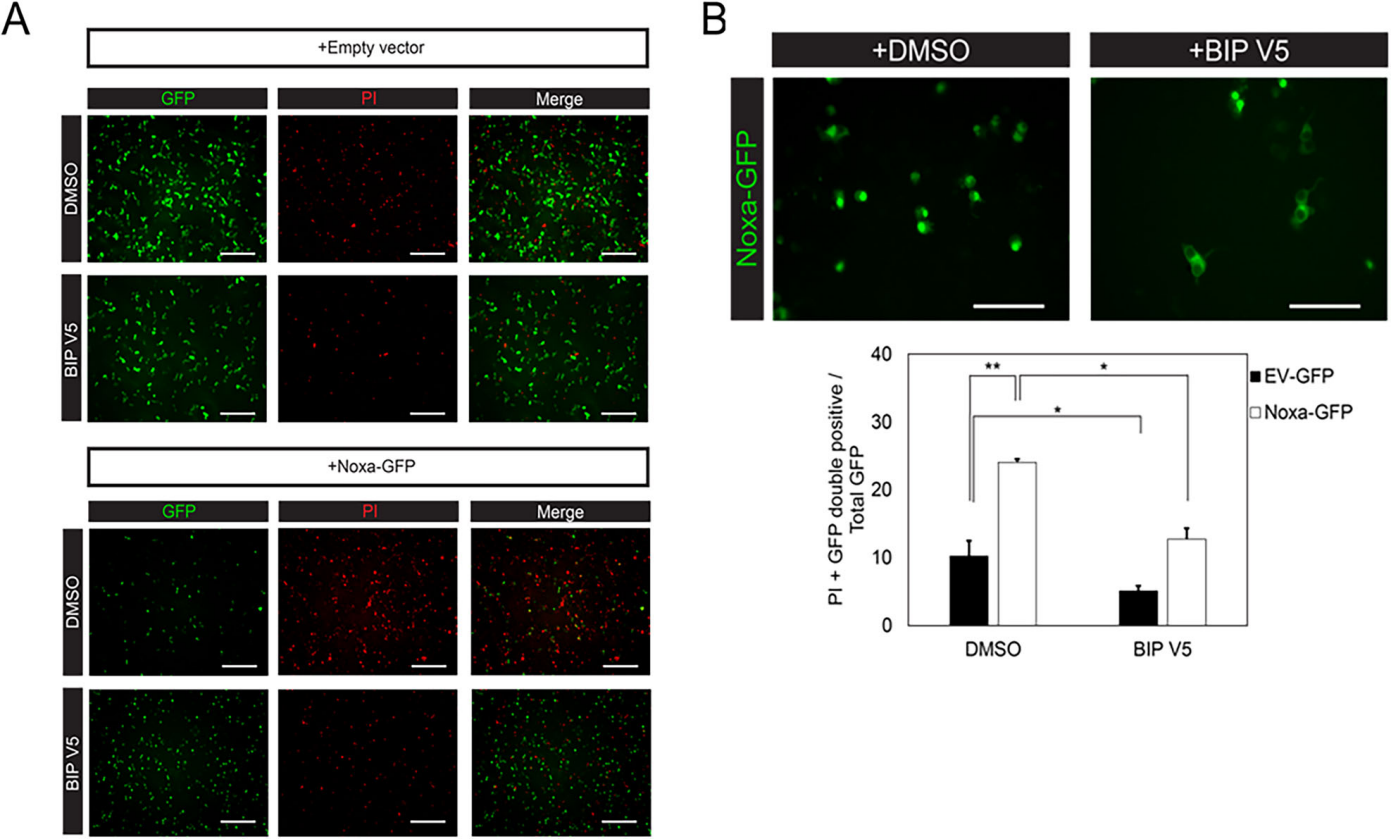
BIP V5 decreases the amount of PI-positive cells in the presence of Noxa. **A** Representative images of MN9D cells overexpressing empty vector-GFP (EV-GFP) or Noxa-GFP with or without pretreatment of BIP V5 and stained for propidium iodide (PI). Scalebars 50 μm*, n* = *3*. **B** Representative images depicting the morphology of Noxa-GFP cells in the absence and presence of BIP V5. Without BIP V5, Noxa-GFP cells display an apoptotic phenotype, whereas in the presence of BIP V5, the cells look normal. Graph: quantification of **A**, normalized for the total amount of cells. Scalebars 10 μm, *n* = 3, *p < 0.05, **p < 0.01.

**Fig. 13:**
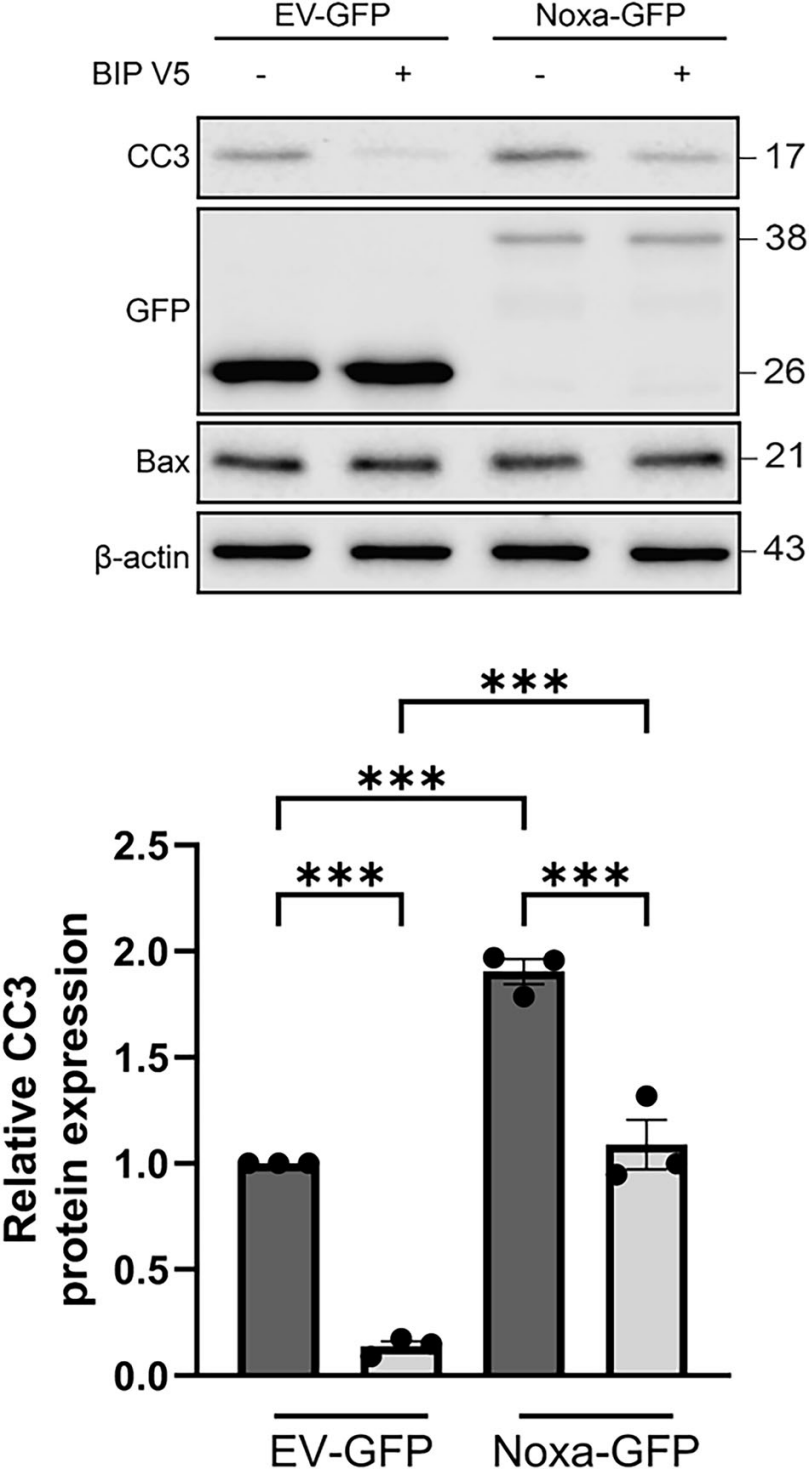
BIP V5 decreases the amount of cleaved caspase 3 in the presence of Noxa. Western blot of MN9D cells overexpressing empty vector-GFP (EV-GFP) or Noxa-GFP with or without pretreatment of BIP V5 *n* = 3; ***p < 0.001.

As *Noxa* expression levels are increased in Pitx3-ablated animals as compared to heterozygous Pitx3 mice, we wondered whether increased Noxa expression is sufficient to induce apoptosis. Therefore, we titrated different concentrations of Noxa together with 100 ng empty vector (EV)-mCherry into dopaminergic MN9D cells, which are dependent on Mcl1 for survival [18]. Noxa overexpression increased CC3 levels significantly at 250, 500 and 1000 ng (100% DMSO control vs 164% ± 10.7 at 250 ng, 243% ± 23.4 at 500 ng and 315% ± 22.7 at 1000 ng; Fig. 9). Moreover, mCherry levels significantly decreased with increasing Noxa concentrations (100% DMSO control vs 67.5% ± 5.0 at 250 ng, 64.3% ± 9.1 at 500 ng and 56.5% ± 3.6 at 1000 ng; Fig. 9). This indeed suggests that Noxa functions as a pro-apoptotic factor in dopaminergic cells, possibly by preventing the anti-apoptotic function of Mcl1. To investigate if indeed Noxa and Mcl1 interact physically, we performed a proximity ligation experiment to visualize this possible interaction. We made use of MN9D cells transfected with human NOXA to perform the PLA as the antibody directed against NOXA did not cross-react with mouse Noxa and was therefore not suited. Indeed, NOXA and Mcl1 clearly interact substantially more over the background in the NOXA-GFP transfected cells (Fig. 10). As the observed cell death could also be related to alteration in Mcl1 protein levels we monitored Mcl1 levels after Noxa-GFP transfection of MN9D cells. Noxa expression did not alter Mcl1 levels but did result in an increase in cleaved caspase 3 as is also shown before (Figs. 9, 11). Previously, we showed that inhibiting the activation and translocation of the pro-apoptotic Bcl2 factor Bax with the Bax-inhibiting peptide V5 (BIP V5) could rescue cells from undergoing apoptosis [18]. Therefore, we assessed whether pretreatment with BIP V5 could protect MN9D cells from undergoing apoptosis in the presence of Noxa. We transiently transfected EV-GFP and Noxa-GFP into MN9D cells with and without BIP V5 pretreatment. Indeed, one-hour pretreatment with 200 μm BIP V5 significantly decreased propidium iodide (PI) staining both basally and in the presence of Noxa (8.4% ± 1.8 DMSO control vs 4.1% ± 0.5 BIP V5, 21.9% ± 0.5 Noxa + DMSO, and 9.1% ± 1.6 Noxa + BIP V5; Fig. 12A). Noxa-GFP cells without BIP V5 pretreatment displayed apoptotic morphology, whereas Noxa-GFP cells with BIP V5 treatment assumed normal morphology (Fig. 12B). Consistent with these results, CC3 levels indeed increased in the presence of Noxa-GFP, but this was rescued by pretreatment with BIP V5 (100% DMSO control vs 14% ± 4.3 BIP V5, 190% ± 10.3 Noxa + DMSO, and 108% ± 20.1 Noxa + BIP V5; Fig. 13). In sum, the data presented above shows that Noxa plays a pro-apoptotic role in dopaminergic cells via the mitochondria-dependent apoptosis pathway, possibly by preventing the anti-apoptotic function of Mcl1, resulting in Bax activation.

## Discussion

### SNc neurons are lost in Pitx3-ablated animals due to a combination of failed embryonic migration and enhanced postnatal elimination

Knocking out Pitx3 results in the loss of Th and other dopamine markers in mdDA neurons during development (starting as early as E12.5) [14], and in the absence of the SNc in the adult midbrain [27, 28]. Here, we show that loss of Pitx3 does not result in cell death during embryogenesis, but rather results in substantial neuronal loss postnatally. Since the loss of mdDA neurons is accompanied by an increase in CC3 (Fig. 6), apoptosis is suggested to be the underlying mechanism. Of note, recently, several non-lethal roles have been ascribed to executor caspases such as CC3, i.e., axon pathfinding and growth cone control (reviewed in [29]). The timepoint of cell death, P3, contradicts previous reports of cell death occurring in the Pitx3-ablated mice at E14.5 [15], this discrepancy, however, might be explained by the very small increase observed in TUNEL-positive neurons at E14.5 (from ~1% to ~2.5% of GFP-positive cells), which would not lead to a significant loss in the total amount of GFP-positive, Pitx3-negative neurons using our approach. Besides a loss of neurons and increased apoptosis postnatally we observed many misplaced neurons in the medial part of the midbrain during embryogenesis. Possibly, apoptosis is the causal mechanism that kills these misplaced cells as they are not in their appropriate niche [10, 11]. In fact, this would further clarify why misplaced mdDA neurons, as well as higher numbers of mdDA neurons in the VTA, were never reported in the adult Pitx3-ablated midbrain.

### Increased *Noxa* expression contributes to the increased amount of cell death in the Pitx3GFP/GFP model

To investigate the mechanism behind the postnatal cell loss in the absence of Pitx3, we focused on the family of Bcl2 proteins. Bcl2 factors play a major role in intrinsic mitochondria-dependent apoptosis by regulating the integrity of the outer mitochondrial membrane (OMM) [30]. The intracellular balance between anti- and pro-apoptotic Bcl2 proteins dictates whether a cell will live or die. Interestingly, we found that the relative expression levels of the BH3-only factor *Noxa* were increased in Pitx3GFP/GFP animals at P2, just before the neurons were lost at P3. Noxa has been shown to preferentially bind to Mcl1 over other anti-apoptotic Bcl2 factors such as Bcl2 and Bcl-xL [26]. Recently, we identified Mcl1 as an important survival factor for adult dopaminergic neurons [18]. The role of Mcl1 in the SN primarily revolves around its function as a pro-survival protein, particularly in the context of dopaminergic neurons.

Besides a role in adult dopamine neurons, Mcl1 has also been shown to be critical during general development as Mcl1 knockouts do not survive the peri-implantation stage [21, 31]. To assess if increased Noxa expression is sufficient to induce cell death we determined the role of Noxa in dopaminergic MN9D cells, which do not express endogenous Noxa and depend on Mcl1 for staying alive. Noxa-induced cell death as visualized by an increase in CC3 and PI-positive cells. Both could be rescued by pretreatment with the Bax-inhibiting peptide BIP V5. Together this suggests that Noxa induces apoptosis through the mitochondria-dependent apoptosis pathway. Indeed, Noxa has been suggested to function via the displacement model, where it binds to Mcl1 and displaces Bak or Bax which is free to translocate to mitochondria to induce MOMP and apoptosis [32]. Interestingly, several studies also propose that the interaction of Noxa with Mcl1 might function as a scaffold for E3 ubiquitin ligases. For example, depletion of NOXA has been proposed to increase MCL1 stability in HeLa cells [25]. Dose-dependent reintroduction of NOXA destabilized MCL1, whereas the introduction of NOXA with a point mutation in the BH3 domain (L92E) failed to rescue MCL1 destabilization after NOXA silencing [25]. In addition, NOXA overexpression in HEK293 cells increases the interaction between MCL1 and the E3 ubiquitin ligase HUWE1, leading to increased Mcl1 degradation [22]. Although we did not observe a decrease in Mcl1 levels, it might still be possible that a Mcl1-specific E3 ubiquitin ligase is not expressed in MN9D cells and that Noxa does function as a scaffold in dopaminergic neurons in vivo. Targeting Noxa or inhibiting the E3-ligase would then favor Mcl1 function and thus increase the apoptotic resilience of the cell [33].

### A role for Pitx3 and Noxa in the adult SNc?

Recently the role of Pitx3 in the maintenance of adult mdDA neurons was investigated. Using a Pitx3 conditional knockout it was demonstrated that Pitx3 deficiency in fully differentiated mdDA neurons results in a rapid reduction in striatal dopamine levels, behavioral abnormalities, and progressive neuronal loss resembling the symptoms of Parkinson’s disease [34]. Pitx3 deficiency resulted in decreased expression of tyrosine hydroxylase (Th) and significant neuronal loss in the SNc, while Th expression remained unaffected in the spared VTA mdDA neurons, likely due to continuous Nurr1 expression [34]. Additionally, a significant increase in cleaved-caspase 3 levels was observed in Pitx3cKO mice, indicating augmented apoptosis of mdDA neurons. The authors link these observations to prior deficiencies in glial cell-derived neurotrophic factor and aldehyde dehydrogenase 1 family member A1 caused by Pitx3 deficiency [34].

**Fig. 14:**
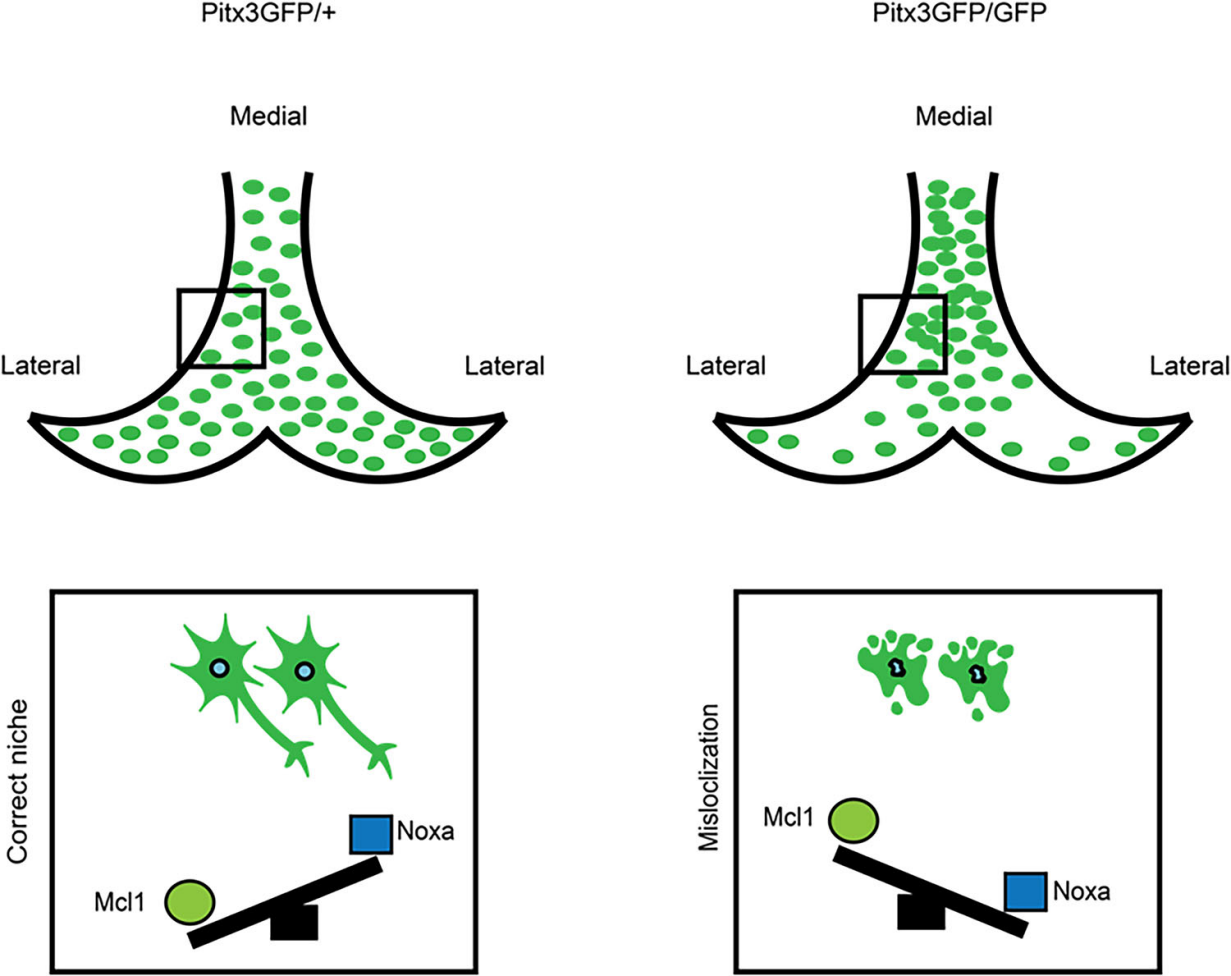
Schematic representation of the development of the SN in the absence of Pitx3. Pitx3GFP/+ heterozygous animals (left) develop normally and cells migrate to their correct niche. The balance between Mcl1 and its specific inhibiting protein Noxa is in favor of Mcl1 allowing cells to survive. In the absence of Pitx3 (right) however, a failure in lateral migration results in more cells in the medial section (mislocalization). In that case, the balance shifts in favor of Noxa, Mcl1 is inhibited and/or degraded, and Bax becomes activated, leading to cell death.

Our study suggests that loss of Pitx3 results in Noxa-dependent apoptosis of dopamine neurons leading to the absence of the SN in the adult (Fig. 14). Although speculative the cell death machinery encompassing Noxa might still be present and functional in the adult and depend on the presence of Pitx3 and as such it would be a crucial pathway to target to attenuate apoptosis and increase resilience of dopamine neurons.

### Concluding remarks and future perspectives

In the substantia nigra dysregulation of apoptotic pathways may have significant implications for neuronal survival and function. Parkinson’s disease (PD), for example, is characterized by the progressive degeneration of dopamine neurons in the substantia nigra, leading to motor impairments and other PD-related symptoms. It is conceivable that dysregulation of Noxa expression or function in the substantia nigra could contribute to the pathogenesis of PD or other neurodegenerative disorders affecting this brain region. However, further research in human post-mortem material is needed to elucidate the specific role of Noxa in the maintenance of the substantia nigra and its implications for neurological health and disease.

## Supplementary information

Supplementary Figure 1

Supplementary Figure 2

Uncropped western blots

## Data Availability

Data may be made available upon reasonable request.

## References

[1] Hegarty SV, Sullivan AM, O’Keeffe GW. Midbrain dopaminergic neurons: a review of the molecular circuitry that regulates their development. Dev Biol. 2013;379:123–38. 23603197 10.1016/j.ydbio.2013.04.014

[2] Veenvliet JV, Smidt MP. Molecular mechanisms of dopaminergic subset specification: fundamental aspects and clinical perspectives. Cell Mol Life Sci. 2014;71:4703–27. 25064061 10.1007/s00018-014-1681-5 PMC11113784

[3] Mesman S, von Oerthel L, Smidt MP. Mesodiencephalic dopaminergic neuronal differentiation does not involve GLI2A-mediated SHH-signaling and is under the direct influence of canonical WNT signaling. PLoS ONE [Internet]. 2014 May [cited 2020 Jan 31]. Available from: https://www.ncbi.nlm.nih.gov/pmc/articles/PMC4035267/. 10.1371/journal.pone.0097926 PMC4035267 24865218

[4] Shults CW, Hashimoto R, Brady RM, Gage FH. Dopaminergic cells align along radial glia in the developing mesencephalon of the rat. Neuroscience. 1990;38:427–36. 1979855 10.1016/0306-4522(90)90039-7

[5] Kawano H, Ohyama K, Kawamura K, Nagatsu I. Migration of dopaminergic neurons in the embryonic mesencephalon of mice. Dev Brain Res. 1995;86:101–13. 7544698 10.1016/0165-3806(95)00018-9

[6] Ohyama K, Kawano H, Asou H, Fukuda T, Oohira A, Uyemura K, et al. Coordinate expression of L1 and 6B4 proteoglycan/phosphacan is correlated with the migration of mesencephalic dopaminergic neurons in mice. Dev Brain Res. 1998;107:219–26. 9593903 10.1016/s0165-3806(97)00220-4

[7] Zhang J, Pho V, Bonasera SJ, Holzmann J, Tang AT, Hellmuth J, et al. Essential function of HIPK2 in TGFβ-dependent survival of midbrain dopamine neurons. Nat Neurosci. 2007;10:77–86. 17159989 10.1038/nn1816 PMC3578579

[8] Oo TF, Burke RE. The time course of developmental cell death in phenotypically defined dopaminergic neurons of the substantia nigra. Dev Brain Res. 1997;98:191–6. 9051260 10.1016/s0165-3806(96)00173-3

[9] Hattori T, McGeer PL. Synaptogenesis in the corpus striatum of infant rat. Exp Neurol. 1973;38:70–9. 4120090 10.1016/0014-4886(73)90008-3

[10] Xu B, Goldman JS, Rymar VV, Forget C, Lo PS, Bull SJ, et al. Critical roles for the netrin receptor deleted in colorectal cancer in dopaminergic neuronal precursor migration, axon guidance, and axon arborization. Neuroscience. 2010;169:932–49. 20493932 10.1016/j.neuroscience.2010.05.025

[11] Van der Heide LP, Smidt MP. The BCL2 code to dopaminergic development and Parkinson’s disease. Trends Mol Med. 2013;19:211–6. 23523055 10.1016/j.molmed.2013.02.003

[12] Smidt MP, van Schaick HS, Lanctôt C, Tremblay JJ, Cox JJ, van der Kleij AA, et al. A homeodomain gene Ptx3 has highly restricted brain expression in mesencephalic dopaminergic neurons. Proc Natl Acad Sci. 1997;94:13305–10. 9371841 10.1073/pnas.94.24.13305 PMC24304

[13] Semina EV, Reiter RS, Murray JC. Isolation of a new homeobox gene belonging to the Pitx/Rieg family: expression during lens development and mapping to the Aphakia region on mouse chromosome 19. Hum Mol Genet. 1997;6:2109–16. 9328475 10.1093/hmg/6.12.2109

[14] Smidt MP, Smits MS, Bouwmeester H, Hamers FPT, van der Linden AJA, Hellemons AJCGM, et al. Early developmental failure of substantia nigra dopamine neurons in mice lacking the homeodomain gene Pitx3. Development. 2004;131:1145–55. 14973278 10.1242/dev.01022

[15] Maxwell SL, Ho HY, Kuehner E, Zhao S, Li M. Pitx3 regulates tyrosine hydroxylase expression in the substantia nigra and identifies a subgroup of mesencephalic dopaminergic progenitor neurons during mouse development. Dev Biol. 2005;282:467–79. 15950611 10.1016/j.ydbio.2005.03.028

[16] van den Munckhof P, Luk KC, Ste-Marie L, Montgomery J, Blanchet PJ, Sadikot AF, et al. Pitx3 is required for motor activity and for survival of a subset of midbrain dopaminergic neurons. Development. 2003;130:2535–42. 12702666 10.1242/dev.00464

[17] Veenvliet JV, Dos Santos MT, Kouwenhoven WM, von Oerthel L, Lim JL, van der Linden AJ, et al. Specification of dopaminergic subsets involves interplay of En1 and Pitx3. Development. 2013;140:3373–84. 23863478 10.1242/dev.094565

[18] Robinson EJ, Aguiar SP, Kouwenhoven WM, Starmans DS, von Oerthel L, Smidt MP, et al. Survival of midbrain dopamine neurons depends on the Bcl2 factor Mcl1. Cell Death Discov. 2018;4:1–13. 10.1038/s41420-018-0125-7 PMC6249233 30479840

[19] Tiklová K, Björklund ÅK, Lahti L, Fiorenzano A, Nolbrant S, Gillberg L, et al. Single-cell RNA sequencing reveals midbrain dopamine neuron diversity emerging during mouse brain development. Nat Commun. 2019;10:581. 30718509 10.1038/s41467-019-08453-1 PMC6362095

[20] Ekholm-Reed S, Baker R, Campos AR, Stouffer D, Henze M, Wolf DA, et al. Reducing Mcl-1 gene dosage induces dopaminergic neuronal loss and motor impairments in Park2 knockout mice. Commun Biol [Internet]. 2019 Apr [cited 2019 Oct 28]. Available from: https://www.ncbi.nlm.nih.gov/pmc/articles/PMC6449387/. 10.1038/s42003-019-0366-x PMC6449387 30963113

[21] Rinkenberger JL, Horning S, Klocke B, Roth K, Korsmeyer SJ. Mcl-1 deficiency results in peri-implantation embryonic lethality. Genes Dev. 2000;14:23–7. 10640272 PMC316347

[22] Gomez-Bougie P, Ménoret E, Juin P, Dousset C, Pellat-Deceunynck C, Amiot M. Noxa controls mule-dependent Mcl-1 ubiquitination through the regulation of the Mcl-1/USP9X interaction. Biochem Biophys Res Commun. 2011;413:460–4. 21907705 10.1016/j.bbrc.2011.08.118

[23] Alves NL, Derks IAM, Berk E, Spijker R, van Lier RAW, Eldering E. The Noxa/Mcl-1 axis regulates susceptibility to apoptosis under glucose limitation in dividing T cells. Immunity. 2006;24:703–16. 16782027 10.1016/j.immuni.2006.03.018

[24] Ploner C, Kofler R, Villunger A. Noxa: at the tip of the balance between life and death. Oncogene. 2008;27:S84–92. 19641509 10.1038/onc.2009.46 PMC3272398

[25] Haschka MD, Soratroi C, Kirschnek S, Häcker G, Hilbe R, Geley S, et al. The NOXA–MCL1–BIM axis defines lifespan on extended mitotic arrest. Nat Commun. 2015;6:1–13. 10.1038/ncomms7891 PMC4423218 25922916

[26] Chen L, Willis SN, Wei A, Smith BJ, Fletcher JI, Hinds MG, et al. Differential targeting of prosurvival Bcl-2 proteins by their BH3-only ligands allows complementary apoptotic function. Mol Cell. 2005;17:393–403. 15694340 10.1016/j.molcel.2004.12.030

[27] Hwang DY, Ardayfio P, Kang UJ, Semina EV, Kim KS. Selective loss of dopaminergic neurons in the substantia nigra of Pitx3-deficient aphakia mice. Mol Brain Res. 2003;114:123–31. 12829322 10.1016/s0169-328x(03)00162-1

[28] Nunes I, Tovmasian LT, Silva RM, Burke RE, Goff SP. Pitx3 is required for development of substantia nigra dopaminergic neurons. Proc Natl Acad Sci USA. 2003;100:4245–50. 12655058 10.1073/pnas.0230529100 PMC153078

[29] Unsain N, Barker PA. New views on the misconstrued: executioner caspases and their diverse non-apoptotic roles. Neuron. 2015;88:461–74. 26539888 10.1016/j.neuron.2015.08.029

[30] Chipuk JE, Moldoveanu T, Llambi F, Parsons MJ, Green DR. The BCL-2 family reunion. Mol Cell. 2010;37:299–310. 20159550 10.1016/j.molcel.2010.01.025 PMC3222298

[31] Arbour N, Vanderluit JL, Grand JNL, Jahani-Asl A, Ruzhynsky VA, Cheung ECC, et al. Mcl-1 is a key regulator of apoptosis during CNS development and after DNA damage. J Neurosci. 2008;28:6068–78. 18550749 10.1523/JNEUROSCI.4940-07.2008 PMC2681190

[32] Willis SN, Chen L, Dewson G, Wei A, Naik E, Fletcher JI, et al. Proapoptotic Bak is sequestered by Mcl-1 and Bcl-xL, but not Bcl-2, until displaced by BH3-only proteins. Genes Dev. 2005;19:1294–305. 15901672 10.1101/gad.1304105 PMC1142553

[33] Robinson EJ, Aguiar S, Smidt MP, van der Heide LP. MCL1 as a therapeutic target in Parkinson’s disease?. Trends Mol Med. 2019;25:1056–65. 31706839 10.1016/j.molmed.2019.08.009

[34] Wang Y, Chen X, Wang Y, Li S, Cai H, Le W. The essential role of transcription factor Pitx3 in preventing mesodiencephalic dopaminergic neurodegeneration and maintaining neuronal subtype identities during aging. Cell Death Dis. 2021;12. 1008. 34707106 10.1038/s41419-021-04319-x PMC8551333

