# Correction: The absence of Pitx3 results in postnatal loss of dopamine neurons and is associated with an increase in the pro-apoptotic Bcl2 factor *Noxa* and cleaved caspase 3

**DOI:** 10.1038/s41419-025-07729-3

**Published:** 2025-06-17

**Authors:** Willemieke M. Kouwenhoven, Edward J. Robinson, Daniek Hamberg, Lars von Oerthel, Marten P. Smidt, Lars P. van der Heide

**Affiliations:** Swammerdam Institute for Life Sciences, Molecular Neuroscience Lab, University of Amsterdam, Amsterdam, The Netherlands

**Keywords:** Differentiation, Neuronal development

Correction to: *Cell Death and Disease* 10.1038/s41419-025-07552-w, published online 1 April 2025

In this article, abstract section line 7, the value “155” should read “15.5”.

The original article has been corrected.

