# Supplementary figures and images for: The absence of Pitx3 results in postnatal loss of dopamine neurons and is associated with an increase in the pro-apoptotic Bcl2 factor Noxa and cleaved caspase 3
Source: Cell Death Dis. 2025 Apr 1;16(1):230. doi: 10.1038/s41419-025-07552-w (PMC11962142; doi:10.1038/s41419-025-07552-w)

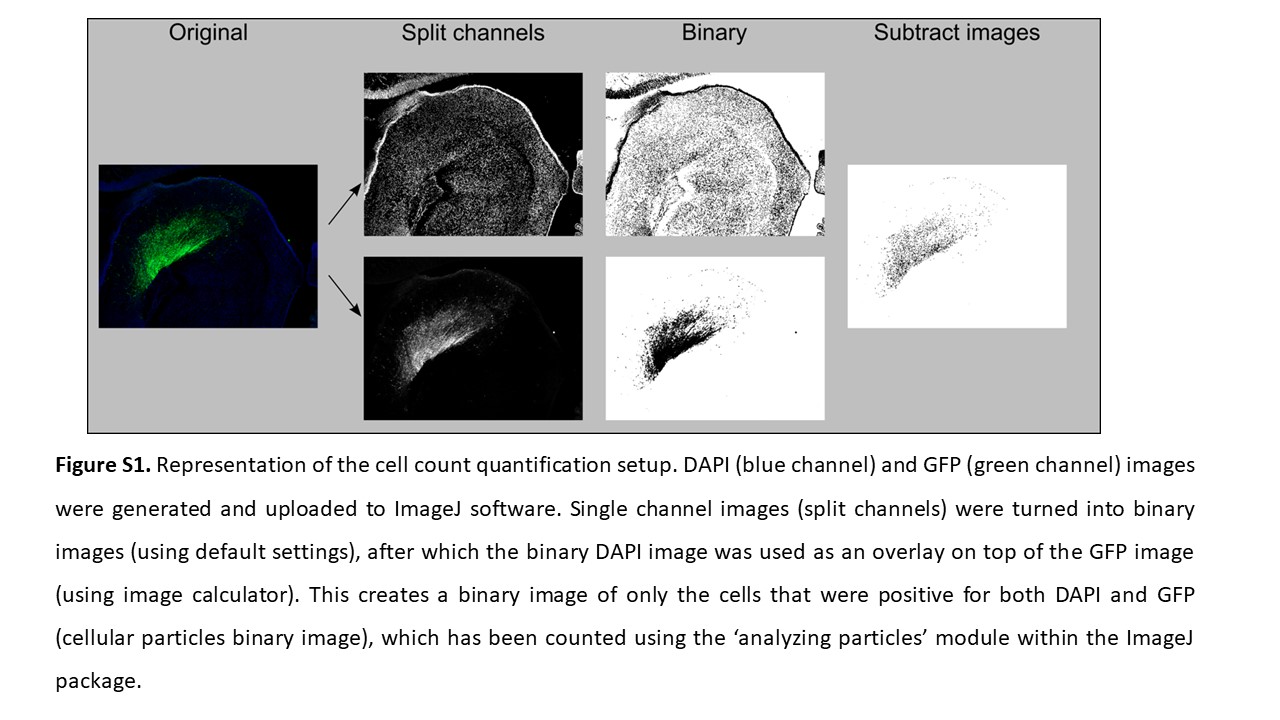

Supplement: Supplementary file 1 — Supplementary Figure 1 [file 41419_2025_7552_MOESM1_ESM.jpg]

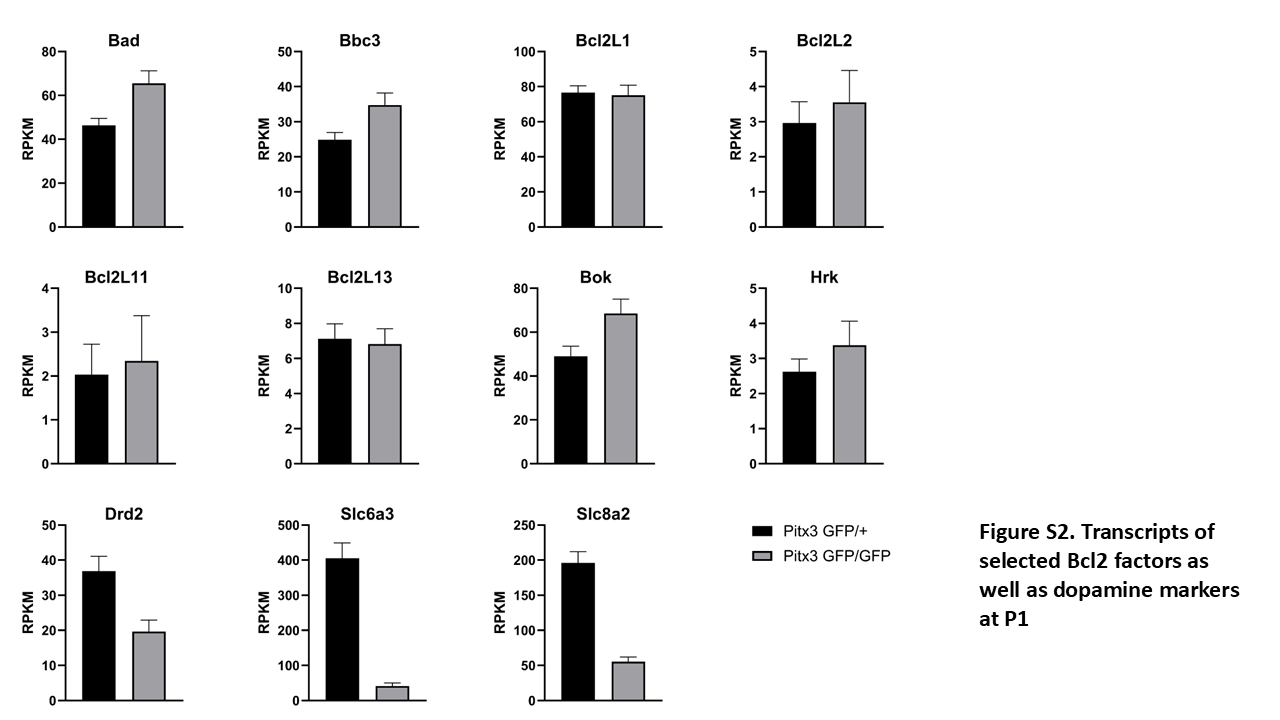

Supplement: Supplementary file 2 — Supplementary Figure 2 [file 41419_2025_7552_MOESM2_ESM.png]
