# Supplementary material for: The absence of Pitx3 results in postnatal loss of dopamine neurons and is associated with an increase in the pro-apoptotic Bcl2 factor Noxa and cleaved caspase 3
Source: Cell Death Dis. 2025 Apr 1;16(1):230. doi: 10.1038/s41419-025-07552-w (PMC11962142; doi:10.1038/s41419-025-07552-w)
Supplement: Supplementary file 3 — Uncropped western blots [file 41419_2025_7552_MOESM3_ESM.pdf]

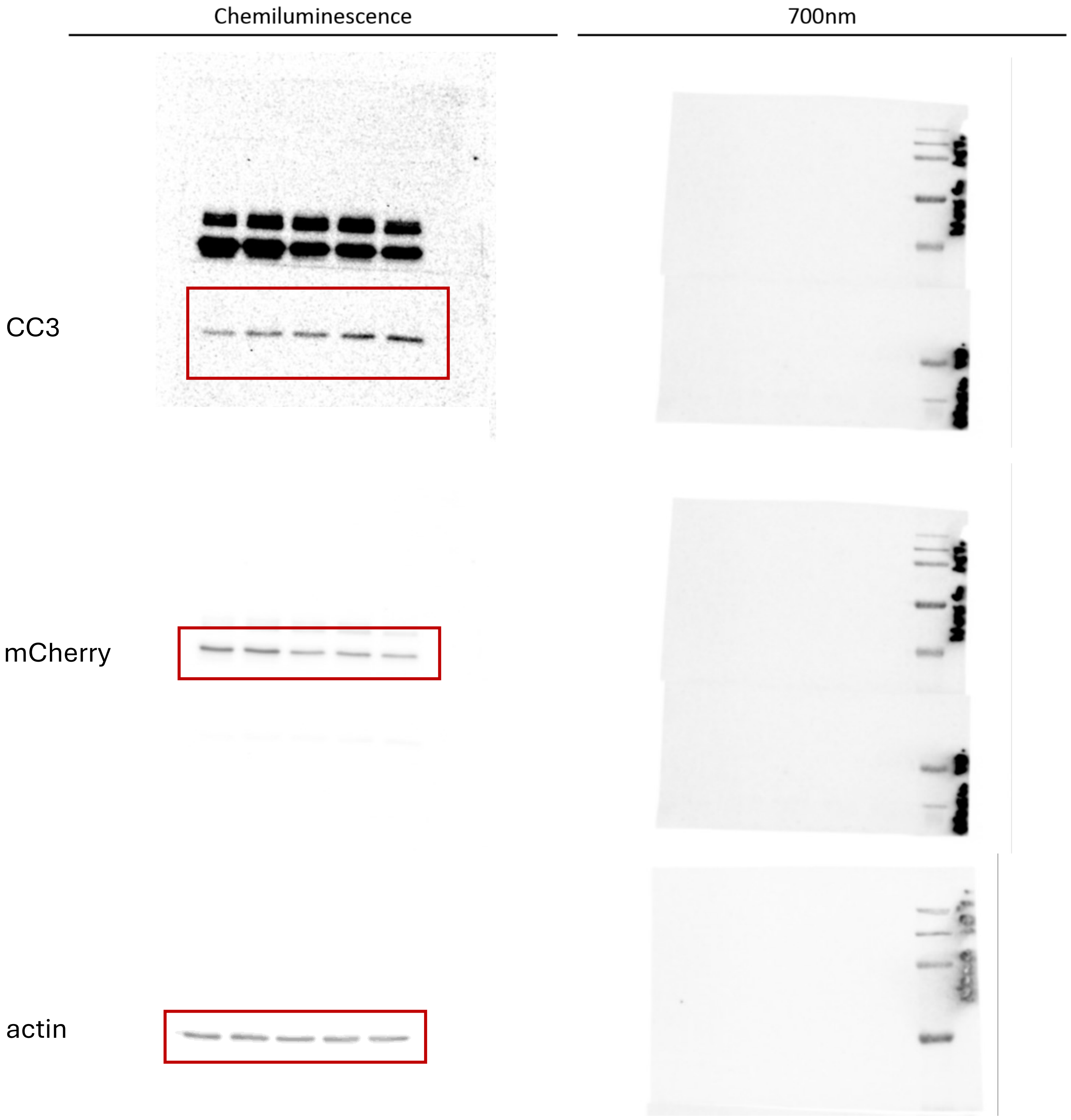
Chemiluminescence
700nm
CC3
mCherry
actin

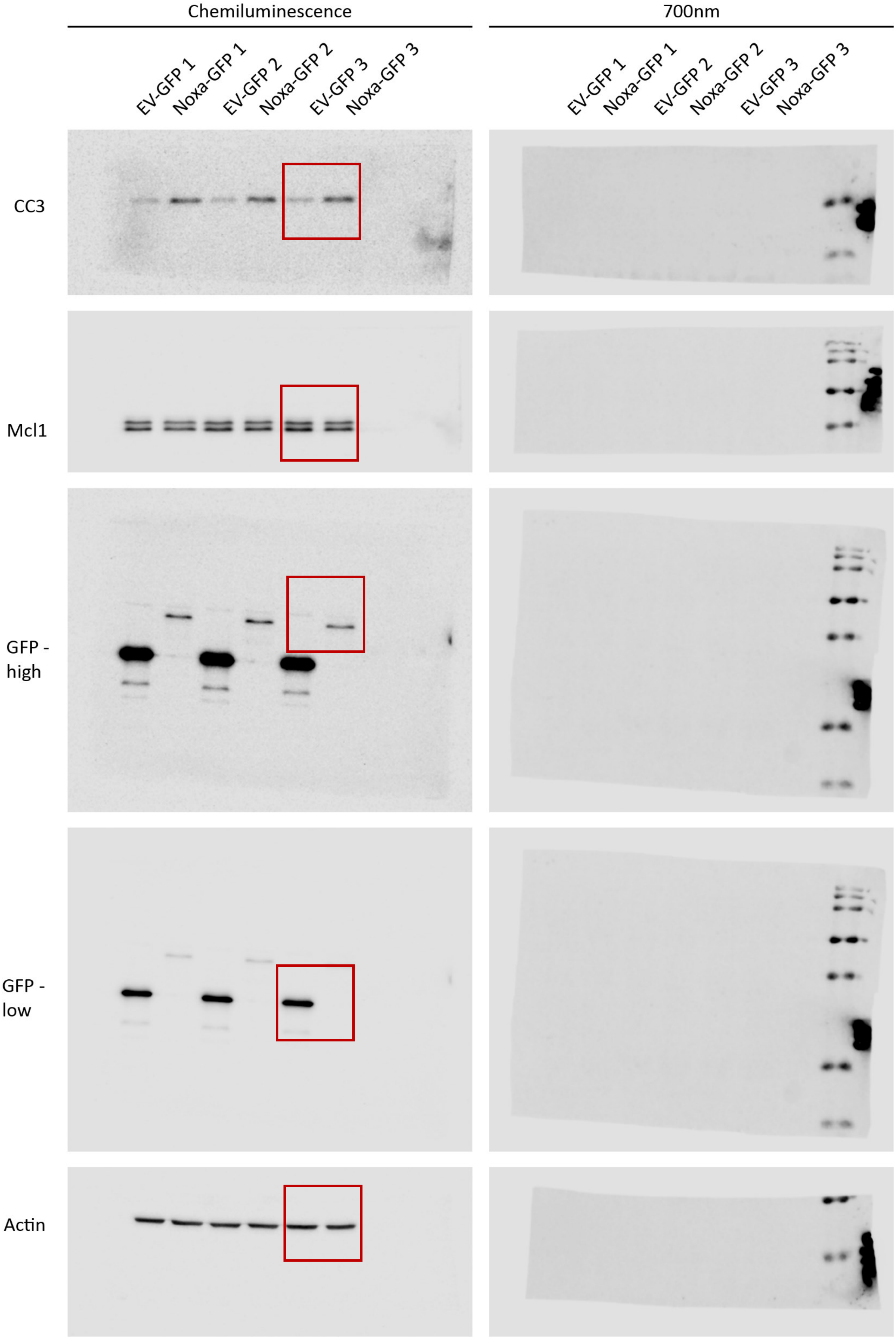
Chemiluminescence
700nm
EV-GFP 1
Noxa-GFP 1
EV-GFP 2
Noxa-GFP 2
EV-GFP 3
Noxa-GFP 3
CC3
Mcl1
GFP - high
GFP - low
Actin

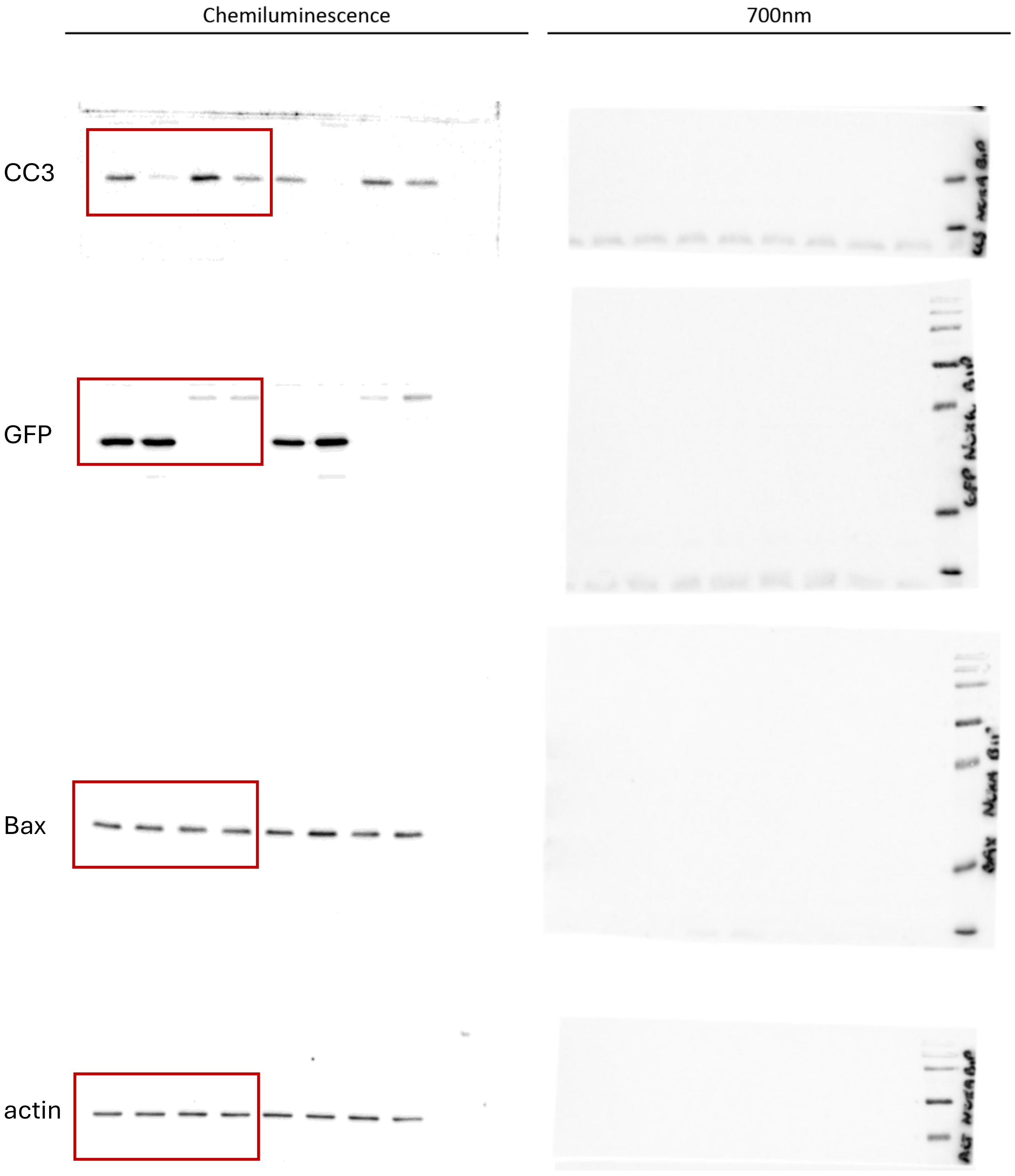
Chemiluminescence
700nm
CC3
GFP
Bax
actin
